# Targeting Plasmids to Limit Acquisition and Transmission of Antimicrobial Resistance

**DOI:** 10.3389/fmicb.2020.00761

**Published:** 2020-05-06

**Authors:** Corneliu Ovidiu Vrancianu, Laura Ioana Popa, Coralia Bleotu, Mariana Carmen Chifiriuc

**Affiliations:** ^1^Microbiology Immunology Department, Faculty of Biology, University of Bucharest, Bucharest, Romania; ^2^The Research Institute of the University of Bucharest, Bucharest, Romania; ^3^The National Institute of Research and Development for Biological Sciences, Bucharest, Romania; ^4^Stefan S. Nicolau Institute of Virology, Bucharest, Romania

**Keywords:** resistance, plasmid curing, infection, antibiotics, CRISPR

## Abstract

Antimicrobial resistance (AMR) is a significant global threat to both public health and the environment. The emergence and expansion of AMR is sustained by the enormous diversity and mobility of antimicrobial resistance genes (ARGs). Different mechanisms of horizontal gene transfer (HGT), including conjugation, transduction, and transformation, have facilitated the accumulation and dissemination of ARGs in Gram-negative and Gram-positive bacteria. This has resulted in the development of multidrug resistance in some bacteria. The most clinically significant ARGs are usually located on different mobile genetic elements (MGEs) that can move intracellularly (between the bacterial chromosome and plasmids) or intercellularly (within the same species or between different species or genera). Resistance plasmids play a central role both in HGT and as support elements for other MGEs, in which ARGs are assembled by transposition and recombination mechanisms. Considering the crucial role of MGEs in the acquisition and transmission of ARGs, a potential strategy to control AMR is to eliminate MGEs. This review discusses current progress on the development of chemical and biological approaches for the elimination of ARG carriers.

## Introduction

The discovery of antibiotics and their clinical use is one of the greatest achievements in medical history. However, the acquisition and dissemination of antimicrobial resistance genes (ARGs) is a severe global problem that emerged in the post-antibiotic era (Friedman et al., 2016; Thaden et al., 2017). The acute limitation of currently available therapeutic options against common infections is responsible for increased rates of morbidity and mortality, longer treatment duration, higher hospitalization costs, and distrust in the efficacy of modern medical practices (Bennett, 2008; Shokoohizadeh et al., 2013; Jiang et al., 2017; Sultan et al., 2018). The most common antimicrobial-resistant bacterial pathogens associated with nosocomial infections were initially gathered under the acronym “ESKAPE” (*Enterococcus faecium*, *Staphylococcus aureus*, *Klebsiella pneumoniae*, *Acinetobacter baumannii*, *Pseudomonas aeruginosa*, and *Enterobacter* species), which was subsequently proposed to be updated to “ESCAPE” (*E. faecium, S. aureus, Clostridium difficile, A. baumannii, P. aeruginosa*, and *Enterobacteriaceae*) (Grundmann et al., 2006; Santajit and Indrawattana, 2016; Penes et al., 2017; World Health Organization [WHO], 2017).

The phenomenon of antimicrobial resistance (AMR) is not new as ARGs have evolved over millions of years (Shlaes et al., 1997). However, AMR is amplified in the presence of the selective pressure exerted by antibiotics (Pelgrift and Friedman, 2013). Between 2000 and 2010, global antibiotic use increased by 36%, and in the case of carbapenems reached 45% (Van Boeckel et al., 2014). Inappropriate use of antibiotics in animals also contributes to rising AMR. The global consumption of antibiotics in animal feed was estimated to be 131,109 tons in 2013, and is expected to reach 200,235 tons in 2030 (Van Boeckel et al., 2017). Incomplete microbe elimination, facilitated by microbiostatic drugs that inhibit multiplication of microbes without killing them, favors the development of drug resistance. Furthermore, incorrect administration of microbicidal drugs in terms of dosing intervals and concentration also contributes to the occurrence of AMR. The dosing interval is essential for antibiotics with a short elimination half-life, such as beta-lactams, tetracyclines, clindamycin, and the majority of macrolides; while concentration is a critical parameter for antibiotics such as vancomycin, aminoglycosides, azalides, ketolides, and quinolones (Gao et al., 2011). Genetic resistance of clinically significant pathogens is amplified by the ability of bacteria to form biofilms on viable tissues or inert substrates; these biofilms exhibit high phenotypic resistance or tolerance to high doses of antimicrobial agents (Giedraitiene et al., 2011). As a result of the selective pressure exerted by antibiotics, bacterial genomes are reshaping, and bacteria adapt and survive in the presence of antibiotics (van Elsas and Bailey, 2002). There are multiple mechanisms of adaptation of resistant bacteria to antibiotics and elucidating these mechanisms will enable the development of effective novel therapies to tackle the increasing threat of resistance.

An important strategy for combating AMR is to diminish the mobilization and persistence of ARGs in bacterial populations. This review highlights current progress in the development of chemical and biological approaches for the elimination of resistance plasmids. Such plasmids play a central role both in horizontal gene transfer (HGT) and as support for other mobile genetic elements (MGEs), in which ARGs are assembled through transposition and recombination mechanisms; the resulting MGEs can then move between chromosomes and plasmids or between plasmids. We will first describe the main MGEs involved in the global dissemination of antibiotic resistance, and then discuss current progress on the development of novel antimicrobial strategies aimed at elimination of MGEs, with a focus on resistance plasmids.

## Role of MGEs in the Acquisition and Transmission of AMR

**FIGURE 1:**
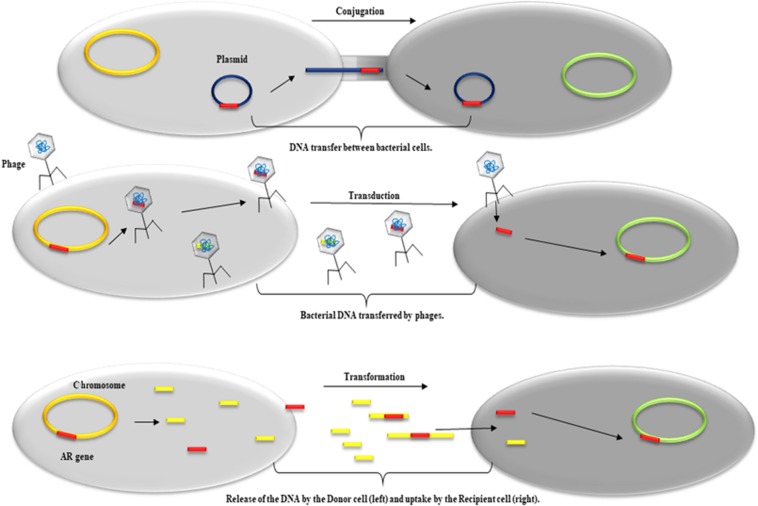
Schematic representation of the predominant HTG mechanisms involved in the acquisition and dissemination of genetic material such as ARG. From top to bottom: *Conjugation*, DNA transfer between a donor cell (left) and a recipient cell (right) mediated by plasmids; *Transduction*, transfer of bacterial DNA between a donor cell (left) and a recipient cell (right) mediated by phages; *Transformation*, release of DNA by a donor cell (left) and uptake by a recipient cell (right).

Resistance to antimicrobials can be acquired through spontaneous mutations in chromosomal genes or by HGT of ARGs. The bacterial genome includes the genomic backbone or core genome, to which a variety of MGEs, termed the accessory genome, is added, and together this comprises the bacterial pan-genome (Guimaraes et al., 2015). The term resistome refers to the part of the pan-genome that contains ARGs, both in commensal and pathogenic bacteria (D’Costa et al., 2006; Landecker, 2016). Many ARGs can move between the bacterial chromosome and plasmids, within the same species or between different species or even genera, through different mobilization mechanisms (conjugation, transduction, and transformation) (Figure 1). HGT is the primary driver of multidrug resistance (MDR) in both Gram-negative and Gram-positive bacteria. MGEs (except for gene cassettes and miniature inverted-repeat transposable elements; MITEs) are DNA fragments encoding enzymes and other proteins that mediate intracellular or intercellular mobility. Intracellular mobility occurs within the same cell, from a chromosome to a plasmid or between plasmids. This type of mobility can be achieved by non-conjugative transposons, gene cassettes, and insertion sequence common region (IS*CR*) elements. These MGEs are mobilized by recombination but can involve replication. Intercellular mobility is achieved by MGE elements that are capable of self-replication and conjugative transfer, such as plasmids and conjugative transposons (Frost et al., 2005; Bennett, 2008; Ilangovan et al., 2015; Partridge et al., 2018). The versatility of MGEs justified the replacement of the constant genome paradigm with that of the fluid genome (Shapiro, 1985; Pinilla-Redondo et al., 2018).

The majority of clinically significant ARGs are located on MGEs. To effectively fight AMR, we need to unravel the role of MGEs in the dissemination of antibiotic resistance among clinically important pathogens.

### Plasmids

Plasmids have a vital role in the accumulation and transfer of ARGs, mainly in Gram-negative bacteria, and are involved in the acquisition of resistance to most antibiotic classes, including β-lactams, aminoglycosides, tetracyclines, chloramphenicol, sulfonamides, trimethoprim, macrolides, polymyxins, and quinolones (Carattoli, 2013; Shintani et al., 2015). Plasmids, either circular or linear, are stable replicons with a complex replication apparatus (Shintani et al., 2015). Generally, plasmids are physically distinct from the primary bacterial chromosome and replicate independently; however, most of the components required for replication are provided by the host (Garcillan-Barcia and de la Cruz, 2008; Guglielmini et al., 2014). Plasmids conferring MDR are usually conjugative, capable of initiating not only their own transfer but also that of other plasmids, and possess mechanisms to control their copy-number in the cell and/or replication ability (Frost et al., 2005; Nordstrom, 2006). Plasmids guarantee transmission through different mechanisms like active partitioning systems, random segregation, or post-segregational killing (Million-Weaver and Camps, 2014). Besides conjugative plasmids, another category of plasmids are mobilizable plasmids, which are smaller in size and not self-transmissible, but they can transfer DNA to a particular host in the presence of conjugative plasmids; this transfer occurs both vertically and by HGT (Bennett, 2008).

The first classification of plasmids was based on incompatibility (*Inc*) groups (the mechanism that prevents the existence of plasmids with the same replication mechanism within the same bacterial cell); specific incompatibility groups were described in *Enterobacteriaceae*, *Pseudomonas* spp., and Gram-positive staphylococci (Frost et al., 2005). In MDR strains of *P. aeruginosa*, a series of plasmids (pS04 90, pBM41, p14057 A, and p14057 B) encoding carbapenemase resistance have been highlighted (Liu et al., 2018; Shi et al., 2018; van der Zee et al., 2018). Strains of *A. baumannii* show plasmid-encoded resistance to carbapenems (Cameranesi et al., 2018; Leungtongkam et al., 2018; Silva et al., 2018), aminoglycosides (*armA*) (Upadhyay et al., 2018), colistin (Jaidane et al., 2018), sulfonamides, or streptomycin (Hamidian et al., 2016). In addition, plasmids conferring resistance to various classes of antibiotics have been found in *Staphylococcus* spp. (Mugnier et al., 2009; Ruiz-Martinez et al., 2011; Hamidian et al., 2016; Holmes et al., 2016; Liu et al., 2016, 2018; Becker et al., 2018; Cameranesi et al., 2018; Fessler et al., 2018; Jaidane et al., 2018; Leungtongkam et al., 2018; Shi et al., 2018; Silva et al., 2018; Upadhyay et al., 2018; van der Zee et al., 2018). Resistance plasmids exhibit a high degree of plasticity, which is translated into an increased frequency of insertions, deletions, and changes in DNA (Kado, 2014). Plasmids may also harbor ARGs encoding efflux pumps that confer an MDR phenotype such as quinolone resistance (Jacoby et al., 2014).

**FIGURE 2:**
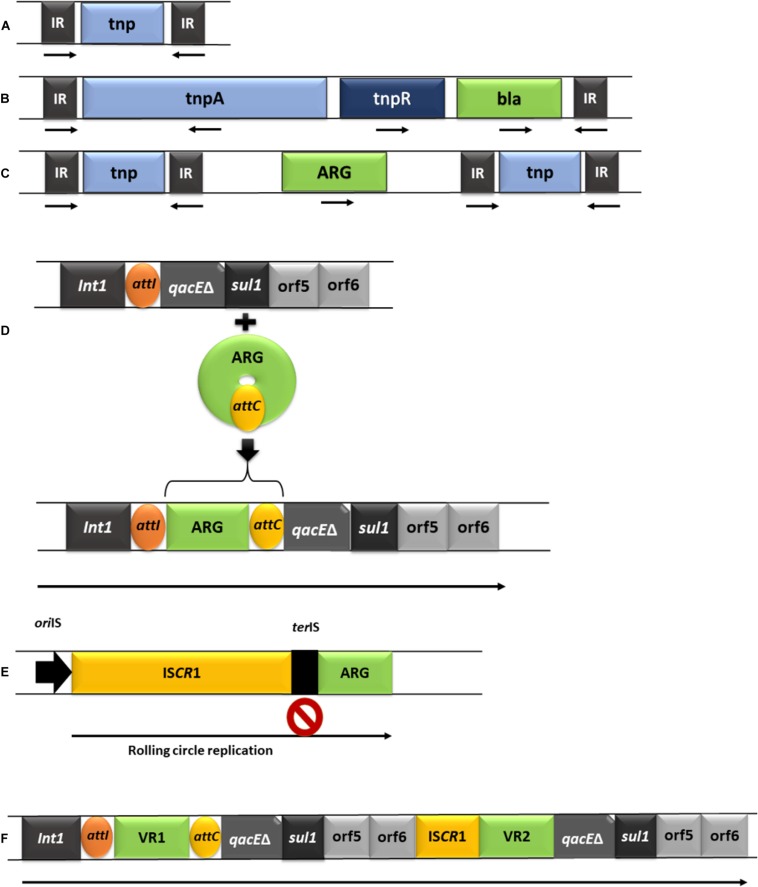
Schematic representation of the predominant MGEs involved in acquisition and dissemination of ARGs. **(A)**, IS element (IR: inverted repeats; *tnp*: transposase gene). **(B)**, Tn*3* complex transposon (*tnp*B: resolvase gene; ARG-antibiotic resistance gene). **(C)**, composite transposon. **(D)**, class I integron and the acquisition of a gene cassette (*Int*1: integrase gene; *att*1: recombination site of the integron; *qac*Eδ: truncated segment belonging to a gene that encodes resistance to quaternary ammonium compounds; sul1: sulfonamide resistance gene; *orf*5/*orf*6: open reading frames, *att*C: recombination site of the gene cassette). **(E)**, the mechanism of acquiring adjacent DNA by ISCR elements (*ori*IS: origin of replication; *ter*IS: end of replication; a second stop sign is located after the ARG, allowing transposition of the entire segment by recombination). **(F)**, complex class 1 integrons (Int1: integrase gene, followed by the *att*I site; VR1/VR2: variable regions e.g., ARGs, followed by the *att*C site).

Further to their direct role in HGT, plasmids can also contribute to the acquisition and dissemination of ARGs to other MGEs in which ARGs are assembled via transposition and recombination mechanisms (Stanisich, 1988; Bennett, 2004, 2008). Some of the MGE frequently involved in the acquisition of clinically relevant ARGs are briefly described below and summarized in Figure 2.

### Insertion Sequences

Insertion sequences (IS) are the smallest (0.7–2.5 Kb) and simplest transposable elements found in bacteria (Mahillon and Chandler, 1998; Aminov, 2011). These elements are usually flanked by short, mostly inverted repeats, which sometimes generate direct target duplications (DR) when they are integrated into the target DNA (Siguier et al., 2015). IS differ from transposons by the absence of cargo or passenger genes, which are responsible for functions other than mobilization. Currently, there are more than 4500 IS listed in dedicated databases like ISFinder (Siguier et al., 2015; Vandecraen et al., 2017). IS are involved in AMR through their ability to transfer ARGs, but also by their ability to modulate the expression of ARGs; this occurs following integration of IS within the ARGs, or by the IS providing an active promoter for ARGs (Siguier et al., 2006; Partridge et al., 2018). For example, IS can increase expression of efflux pumps (Olliver et al., 2005; Siguier et al., 2015). The role of IS in antibiotic resistance has been highlighted by numerous studies, particularly those related to resistance to colistin and carbapenem. The most common mechanism for the development of colistin resistance is inactivation of the gene *mgrB* in *K. pneumoniae*, following the transposition of different types of IS, such as IS903, ISKpn26, IS10R, and IS5 (Cannatelli et al., 2014; Berglund et al., 2018). In colistin-resistant strains of *Klebsiella* sp., alteration of *mgrB* and *phoP* gene sequences by different IS, such as ISKpn14, ISKpn28, IS903, IS5, and IS3, can sometimes induce a pandrug-resistance phenotype (Giordano et al., 2018; Uz Zaman et al., 2018). IS also play a vital role in carbapenem resistance through a mechanism similar to that of colistin resistance but involving the inactivation of *oprD* and *omp* genes (Lev et al., 2017; Bocharova et al., 2019). The *oprD* gene is inactivated by the insertion of ISPpu-21 (Shariati et al., 2018). In addition to the IS themselves, there are other similar transposable elements (TEs) that harbor transposase genes (autonomous) or depend on host cell elements (non-autonomous) (Siguier et al., 2015). When IS elements are carrying passenger genes, they are termed IS transporters (tISs) (Siguier et al., 2006). In contrast to complex transposons that exist only as a single copy in a specific replicon, IS can be present as multiple copies, thus contributing to the accumulation of ARGs (Rankin et al., 2011).

### Resistance Transposons

Transposons (Tn) are a category of MGEs that carry ARGs. Many Tn have the ability to jump from/to different locations in the genome, and are capable of mediating the mobility of both intramolecular and intermolecular ARG (Bennett, 2004, 2008; Babakhani and Oloomi, 2018). Bacterial Tn can be divided into two types, composite (two IS elements flanking a central gene) and complex (containing the *tnpA* gene encoding transposase, the *tnpR* gene encoding resolvase, as well as one or more cargo genes) (Genilloud et al., 1988; Bennett, 2008; Partridge, 2011). MITEs and palindrome-associated transposable elements (PATEs) are included in the category of non-autonomous derivatives (Siguier et al., 2015). The predominant ARG-containing Tn whose transmission is a challenge when treating infections are Tn5 (encoding resistance to neomycin and kanamycin in *A. baumannii* and *P. aeruginosa*), Tn10 (encoding tetracycline resistance), Tn9, Tn903, Tn1525, and Tn2350 (Genilloud et al., 1988; Partridge, 2011).

### Integrons

Integrons are MGEs that have the ability to accumulate gene cassettes, including ARGs, and to disseminate them through other MGEs. Sedentary integrons are DNA elements found in the chromosomes of many species and were initially discovered due to their association with AMR (Mazel, 2006; Partridge et al., 2009). In contrast to sedentary integrons, mobile resistance integrons are often found in plasmids (Ponce-Rivas et al., 2012). The role of these elements in the acquisition and dissemination of ARGs is crucial, especially in Gram-negative bacteria (Ponce-Rivas et al., 2012), but they are also present in Gram-positive bacteria (Nandi et al., 2004). Integrons contain the gene encoding integrase (*IntI*), an enzyme that allows the incorporation of circular DNA segments by site-specific recombination (Cambray et al., 2010). They also harbor a specific integration site, at which one or more gene cassettes can be inserted by the integrase (Recchia and Hall, 1995; Bennett, 2008; Partridge et al., 2009). Gene cassettes are usually small DNA fragments of 500–1000 base pairs, which can be mobilized by integrase. Generally, the gene cassettes comprise a single open reading frame (ORF) followed by a short recombination site termed *attC* (formerly “59 bases element”). Since the majority of these cassettes are promoterless, expression of their genes depends on the integron promoter (Bennett, 1999, 2008). Gene cassettes contain ARGs encoding resistance to different antibiotic classes (Recchia and Hall, 1995; Nordmann and Poirel, 2002), as well as antiseptics and disinfectants (Recchia and Hall, 1995; Bennett, 2008). Integrons are divided into several classes (class 1, class 2, and class 3) depending on the amino acid sequence of the *IntI* enzyme. Class 1 integrons, which are typically associated with plasmids, are most commonly encountered in clinical isolates from hospitals and elderly care facilities, but have also been found in food production chain isolates (e.g., cattle farm isolates) (Belaynehe et al., 2018; Faghri et al., 2018; Rajpara et al., 2018). IS*CRs* are transposable elements that are a similar size to IS elements, are often associated with class 1 integrons, and are capable of mobilizing adjacent DNA via a rolling-circle mechanism (Bennett, 2008). When IS*CR* elements are associated with class 1 integrons, they form complex class 1 integrons (Bennett, 2008; Toleman and Walsh, 2008, 2011).

### Genomic Islands

In addition to classical MGEs, such as conjugative plasmids or resistance transposons, an additional category of MGEs is a series of genomic islands that are capable of mediating their own excision, called integrative and conjugative elements (ICE) (Burrus et al., 2002; Burrus and Waldor, 2004; Dobrindt et al., 2004; Juhas et al., 2009; Wozniak and Waldor, 2010). The concept of pathogenicity islands (PAIs) was first described in 1980 by Hacker et al. (1983) who analyzed the virulence mechanisms of strains of *E. coli* isolated from urine cultures and observed the presence of unstable chromosomal regions bearing different virulence characteristics. Studies on multiple genomic islands have identified several common and essential features of these chromosomal regions: they are DNA segments with a size of 10–200 kb; they insert within tRNA genes; they contain directly repeated recognition sequences; and they contain cryptic genes encoding factors involved in integration, insertion, or transfer (Hacker et al., 1990).

### Integrative and Conjugative Elements

Integrative and conjugative elements (ICE) were first described in 1946 by Lederberg and Tatum (1946), and are responsible for HGT of most resistance and virulence factors (Llosa et al., 2002; Burrus and Waldor, 2004; Fernandez-Lopez et al., 2006; de la Cruz et al., 2010; Smillie et al., 2010). ICE are 18–600 kbp in size and share several common characteristics with genomic islands, including insertion at a specific site, association with phage integrase genes, and being flanked by inverted repeats (Toleman and Walsh, 2011). Excision and integration of ICE are accomplished through a recombinase, often termed an integrase. The integrases associated with ICE are tyrosine or serine recombinases, and are homologous to the integrases found in temperate phages (Wozniak and Waldor, 2010). The insertion site for ICE in the bacterial chromosome is *attB* and is usually located in the gene encoding tRNA, hence the ICE attachment site is termed *att* (Grindley et al., 2006). Some ICE have low specificity for the *att* site, and thus may have an affinity for other sites (Bedzyk et al., 1992; Roberts and Mullany, 2009). Similar to conjugative plasmids, the excision and transfer of ICE are mediated by a type IV secretion system, but in contrast to the conjugative plasmids, which are capable of autonomous replication, ICE integrate into the chromosome and replicate with it (Burrus, 2017). However, some ICE are capable of autonomous plasmid-like replication (Johnson and Grossman, 2015). When ICE are mobilizing bacterial DNA, such as genomic islands, they are termed integrative mobilization elements (IME) (Gonzalez-Candelas and Francino, 2012). In terms of ICE conjugation, the transfer mechanism is similar to that encountered in plasmids. In the case of plasmids, the relaxase enzyme binds to the DNA and introduces a break in *oriT* to initiate rolling-circle replication. Relaxase remains bound to the single-stranded DNA and forms a complex with a specific coupling protein that allows the translocation of DNA into the recipient cell (Lanka and Wilkins, 1995). ICE mediate the acquisition of genes conferring selective advantages such as resistance to antibiotics or heavy metals, degradation of some compounds, increased bacterial fitness, ability to achieve symbiosis, use of alternative carbon sources, expression of virulence factors such as type III and IV secretion systems, which play an essential role in regulating contact with host cells, disruption of signal transduction, or promotion of apoptosis (Roberts and Smith, 1980; Shoemaker et al., 1980; Mays et al., 1982; Magot, 1983; Hochhut et al., 1997; Ravatn et al., 1998; Nishi et al., 2000; Dobrindt et al., 2004; Schmidt and Hensel, 2004).

Integrative and conjugative elements play a vital role in the acquisition and intercellular transmission of ARGs. Through their own integration and mobilization apparatus, these elements have the ability to mobilize adjacent sequences, including genomic islands or composite transposons carrying ARGs (Delavat et al., 2017). Examples include Tn*10* found in ICEHpaT3T1 from *Haemophilus parainfluenzae* and ICEHin1056 from *H. influenzae*, containing tetracycline and chloramphenicol resistance genes (Juhas et al., 2007); R391, a plasmid of the SXT ICE family that carries kanamycin resistance genes (Pembroke et al., 2002), and ICEPmiJpn1 described in *Proteus mirabilis* and encoding resistance to broad-spectrum beta-lactamases (Harada et al., 2010; Mata et al., 2011). There are also a number of ICE encountered in *H. influenzae* (ICEHin1056, ICEHin299, ICEHin2866, ICEHpa8f, ICEHin028, ICEHinB) (Juhas et al., 2007). ICEEc2, identified in *E. coli*, contains Tn*7*, which can be mobilized independently, and class 2 integrons. Tn*7* carries *dfrA1*, *sat2*, and *aadA1*, which are responsible for resistance to trimethoprim, streptothricin, and streptomycin/spectinomycin, respectively (Roche et al., 2010). Another large ICE family is ICE*Tn4371* found in Beta- and Gamma-Proteobacteria. Members of this ICE family, such as ICE*_*Tn4371*_*6061 found in *P. aeruginosa*, display transfer mechanisms similar to IncP plasmids and carry different ARGs (Castanheira et al., 2007). The Tn*21* transposon subfamily, containing pKLC102/PAPI-1 and PAGI-2/PAGI-3 (*P. aeruginosa*-pathogenicity island-type ICE) carbapenem resistance genes are integrated into tRNA^*Lys*^ and tRNA^*Gly*^ (Klockgether et al., 2007). The Tn*4371* family in *P. aeruginosa* (e.g., ICE_*Tn*_*_4371_*6061) also carries carbapenem ARGs (Fonseca et al., 2015). Tn*916*-type elements that encode tetracycline or minocycline resistance, via the *tet*(M) gene, may embed additional ARGs for other antibiotics such as macrolides, lincosamides, and streptogramins (MLS) and kanamycin/neomycin in the case of Tn*1545* (Cochetti et al., 2008). Roberts et al. (2008) observed that the majority of transposable elements, including composite transposons, mobilizable transposons, ICE, and genomic islands, possess similar transposition mechanisms (serine or tyrosine recombinases). Consequently, it was suggested that all these elements capable of integration and conjugation should be called conjugative transposons, even though most of them integrate into a single specific site (Roberts et al., 2008). The term “conjugative transposon” was first used by Franke and Clewell (1981) when characterizing the Tn*916* element from *E. faecalis* (Franke and Clewell, 1981; Brochet et al., 2009).

## Targeting MGEs to Combat Antibiotic Resistance

The ability of bacteria to adapt to all currently available antibiotics has led to an acute need for new, more effective antibiotics or the development of alternative therapeutic strategies (Seal et al., 2018). MGEs, especially those containing resistance plasmids, transposons, and integrons, play a crucial role in the accumulation and dissemination of ARGs in both the clinical and environmental sectors. Consequently, there is a strong argument for considering that one potential strategy to control AMR is through the elimination of these MGEs. In the field of medicine, the concept of “curing” refers to various clinical techniques applied to repair a defective system (Kennedy, 1981; Dow, 1990). In terms of AMR, “curing” is predominantly used to describe the process of removing ARGs from bacterial populations, and compounds used for this purpose are called “curing agents.” Considering that most ARGs and virulence factors are located on plasmids, the term “curing” has been associated with the removal of plasmids since 1971 (Bouanchaud and Chabbert, 1971). Over the past half-century, several studies have focused on testing antibacterial compounds, such as detergents, biocides, intercalary agents, and nanoparticles (Table 1), bacteriophage- and microbiota-based therapies, or the CRISPR system for curing resistance plasmids (Buckner et al., 2018).

**TABLE 1:** Plasmid curing compounds.

Curing agent	Species of interest	Plasmid target	References
SDS	*S. aureus*	Penicillinase plasmid	Sonstein and Baldwin, 1972
	*E. coli*	pBR322;pBR325	Keyhani et al., 2006
	*E. coli*	212587, 212973, 208366, and 207940 isolates carrying plasmids	Zaman et al., 2010
	*P. aeruginosa*	pBC15 plasmid	Raja and Selvam, 2009
Ethidium bromide	*Streptomycetes*	pIJ303 and pIJ61 plasmids	Crameri et al., 1986
	*E. coli*	212587, 212973, 208366, and 207940 isolates carrying plasmids	Zaman et al., 2010
	*L. acidophilus*	20.3 bp chloramphenicol resistant plasmid	Karthikeyan and Santosh, 2010
	*E. aerogenes*	pKpQIL carbapenem resistant plasmid	Pulcrano et al., 2016
Acridin-orange Acriflavine	*Salmonella*	Chloramphenicol resistant plasmid	Adetosoye and Rotilu, 1985
	*Shigella*		
	*Lactobacillus*	pDR101	Chassy et al., 1978
	*O. oeni*	pRS1, pRS2, and pRS3	Mesas et al., 2004
	*E. coli*	212587, 212973, 208366, and 207940 isolates carrying plasmids	Zaman et al., 2010
	*S. aureus*	Beta-lactam resistance plasmid	Ojo et al., 2014
Triclosan (irgasan) Fusidic acid	*S. aureus*	Mupirocin resistance 48 Md plasmid	Irish et al., 1998
	*E. coli*	pMIB4 plasmid	Riber et al., 2016
Nitric oxide nanoparticles	*P. aeruginosa*	Plasmid carrying antibiotic-resistance genes	Jones et al., 2010
	*S. aureus*		
	*E. coli*		
	*Trichophyton mentagrophytes*		
	*T. rubrum*		
	*A. baumannii*		
Chitosan	*E. coli*	Plasmid carrying antibiotic-resistance genes	Bavya et al., 2019
	*S. aureus*		
Silver nanoparticles	*S. aureus*	methicillin-resistant plasmid	Huang et al., 2011

### Chemical Strategies for Removing MGEs

Chemical agents used for the elimination of resistance elements in bacteria act through several mechanisms, including replication blockage, DNA breaks, or inhibition of conjugation (Tables 1, 2). The effectiveness of the agent varies depending on the bacterial strain, presence of plasmids, and growth conditions.

**TABLE 2:** Conjugation inhibitors and their targets for the elimination of antibiotic resistance.

Conjugative inhibitor	Species of interest	Target	Results	References
Intercalating agents	*Salmonella typhimurium*	Plasmid resistance determinants	Inhibition of plasmid DNA replicons	Hahn and Ciak, 1976; Adetosoye and Rotilu, 1985
Nitrofuran derivatives	*Enterobacteriaceae*	Plasmid DNA replication	DNA replication blocking	Michel-Briand and Laporte, 1985
Unsaturated fatty acids	*E. coli*	R388 and the F-plasmid derivative pOX38	Plasmid conjugation inhibition	Fernandez-Lopez et al., 2005
Bisphosphonates	*E. coli*	Relaxase enzyme	Disrupting conjugative DNA transfer	Lujan et al., 2007
Antibodies	*E. coli*	Relaxase activity	Relaxase blocking, inhibition of conjugative transfer	Garcillan-Barcia et al., 2007
Chemical inhibitors of transposons recombination	*E. coli*	Tn3 recombinase	Tn3 transposition blocking	Fennewald and Capobianco, 1984

Detergents have been used to remove resistance plasmids since 1972. Sodium dodecyl sulfate (SDS) has shown excellent efficiency in removing resistance plasmids in both Gram-positive (e.g., the penicillin resistance plasmid from *S. aureus*) and Gram-negative (e.g., *E. coli* and *P. aeruginosa*) bacteria (Sonstein and Baldwin, 1972; Keyhani et al., 2006; Raja and Selvam, 2009; Zaman et al., 2010). However, high concentrations of SDS are required, which result in gastrointestinal side effects and thus prohibit the use of SDS in humans and animals (Buckner et al., 2018). Another class of compounds used to remove resistance plasmids are the intercalating agents, such as ethidium bromide, acridine-orange, and acriflavine. Elimination of resistance plasmids by ethidium bromide has been demonstrated in Gram-positive (*Lactobacillus acidophilus*) and Gram-negative (*E. coli, Enterobacter aerogenes*) bacteria, as well as in actinomycetes (*Streptomycetes*) (Crameri et al., 1986; Karthikeyan and Santosh, 2010; Zaman et al., 2010; Pulcrano et al., 2016). Acridine-orange and acriflavine have successfully cured resistance plasmids in *E. coli* (Zaman et al., 2010), *Salmonella* spp. and *Shigella* spp. (Adetosoye and Rotilu, 1985), *Lactobacillus* spp. (Chassy et al., 1978), *Oenococcus oeni* (Mesas et al., 2004), and *S. aureus* (Ojo et al., 2014). However, the use of intercalating agents is associated with the risk of mutagenic effects. Furthermore, intercalating agents appear to be inefficient in eliminating large plasmids, such as those found in *Rhizobium* spp. and *Agrobacterium* spp. (Rosenberg et al., 1981). Biocides such as triclosan (irgasan) or fusidic acid have been used since 1998 for the successful removal of resistance plasmids in Gram-negative (*E. coli*) and Gram-positive (methicillin-resistant *S. aureus*) bacteria (Irish et al., 1998; Riber et al., 2016).

Recently, nanoparticles have been proposed as potential tools to combat bacterial resistance (Jones et al., 2010; Bavya et al., 2019). An advantage of using nanoparticles is that they simultaneously target multiple structures, decreasing the risk of selecting/acquiring resistance to them (Zhang et al., 2010). Nanoparticles exert their antibacterial effects through multiple mechanisms, including destruction of the bacterial membrane with elimination of cytoplasmic components, inactivation of DNA or protein binding, and release of reactive oxygen species. Blocking the function of cellular components leads to oxidative stress, electrolyte imbalance, enzyme inhibition, and finally, cell death (Huang et al., 2011; Knetsch and Koole, 2011; Wang et al., 2017). Due to their effects on DNA integrity (double-strand breaks, deaminations, alkylating agent formation, and inhibition of DNA repair enzymes) (Schairer et al., 2012; Nejdl et al., 2017), different nanoparticles might also be regarded as MGE curing agents. Another advantage of using nanoparticles is that their antibacterial action can be maintained for an extended period of time with no loss in stability (Cheow and Hadinoto, 2014). Platinum and copper nanoparticles are instrumental in the elimination of resistance plasmids as they interact with the supercoiled plasmid DNA or with topoisomerases involved in replication, transcription, and recombination processes, ultimately leading to elimination of the plasmids (Lakshmi et al., 1988; Lakshmi and Polasa, 1991; Antonoglou et al., 2019). Copper nanoparticles have also been used in plasmid DNA degradation experiments as well as for blocking plasmid conjugation (Chatterjee et al., 2014; Klumper et al., 2017). Despite the potential for metal nanoparticles to be used as weapons against AMR, bacteria are capable of developing resistance to the nanoparticles themselves, probably facilitated by the global use of metals in fields such as agriculture, animal feed supplements, and disinfectant production (Gupta and Silver, 1998; Rai et al., 2012). Bacterial resistance to silver, copper, and zinc nanoparticles has been highlighted in both Gram-negative and Gram-positive bacteria isolated from inert surfaces, soil, or the intestinal contents of animals fed with zinc and copper supplements (Cason et al., 1966; Santo et al., 2010; Altimira et al., 2012; Yazdankhah et al., 2014; Poole, 2017; Xu et al., 2017). Genes conferring resistance to metals are usually located on plasmids, posing a significant risk of very rapid dissemination through HGT (Dupont et al., 2011). Furthermore, the co-existence of antibiotic resistance and metal resistance genes within the same MGE is a possible mechanism for selecting antibiotic resistance (Poole, 2017). Another type of nanoparticle, the organic nanoparticles such as chitosan nanoparticles, demonstrate resistance curing activity by affecting the integrity of plasmid DNA and the conjugation capacity (Bozkir and Saka, 2004).

Conjugation is involved in the dissemination of plasmids and other MGEs such as conjugative transposons and ICE. Since most MGEs use the same proteins for their transfer, HGT could be blocked by conjugation inhibitors (COINs) (de la Cruz et al., 2010; Baquero et al., 2011; Table 1). Many experiments have demonstrated that intercalating agents, heterocyclic compounds, acridine dyes, quinolones, and unsaturated fatty acids such as linoleic and linolenic acid can act as COINs (Hahn and Ciak, 1976; Adetosoye and Rotilu, 1985; Michel-Briand and Laporte, 1985; Molnar et al., 1992; Fernandez-Lopez et al., 2005). A possible target of COINs is relaxase, the most critical enzyme in the conjugation process as it cuts the plasmid at the *oriT* origin. Inhibiting conjugation by targeting relaxase has been demonstrated for bisphosphonates (etidronate, clodronate) and for specifically designed antibodies (Garcillan-Barcia et al., 2007; Lujan et al., 2007). Another potential target of COINs is to limit or block the site-specific recombinase enzymes that have a central role in the transposition process (Fennewald and Capobianco, 1984). However, some COINs are unable to translocate the bacterial cell membrane, hence research has been directed toward the discovery of new classes of permeable compounds (Wigle et al., 2009).

Chemical agents have been successfully used to remove resistance plasmids, but use of these compounds to limit antibiotic resistance in humans is problematic. As stated previously, high concentrations of SDS are required to remove resistance plasmids and this results in gastrointestinal side effects such as colitis; consequently, SDS use is banned in humans and animals (Raja and Selvam, 2009). Studies have demonstrated that intercalating agents are effective in eliminating resistance plasmids (Chassy et al., 1978; Mesas et al., 2004; Ojo et al., 2014; Pulcrano et al., 2016), but the risk of mutagenic effects must be considered. Nanoparticles are another weapon against bacterial resistance, but the main impediment to their use in humans is the lack of information regarding their safety and how they affect the biological integrity of organisms, particularly in terms of producing toxicological, cytotoxic, and genotoxic effects (Li T. et al., 2018). Nanoparticles are predominantly used in doses below the threshold concentrations; thus, they are not considered harmful to the body. However, bioaccumulation of nanoparticles in the body following long-term exposure is well known (Hasan et al., 2018). Therefore, further research on long-term nanoparticle toxicity and carcinogenesis is needed. Quinolones inhibit bacterial DNA replication by interfering with DNA-gyrase activity, and numerous studies have highlighted the plasmid-curing effect of quinolones in *E. coli in vitro* and *in vivo* assays (Weisser and Wiedemann, 1985; Michel-Briand et al., 1986; Courtright et al., 1988; Fu et al., 1988; Selan et al., 1988). Despite these results, the use of quinolones to eliminate plasmids containing ARGs may lead to a fitness advantage in plasmid-containing cells and would therefore select for plasmid maintenance. Phenothiazines, such as chlorpromazine, also have plasmid-curing activity in *E. coli* (Molnar et al., 1976) and methicillin-resistant *S. aureus* (Costa et al., 2010). Although the role of these chemical agents has been demonstrated *in vitro*, further studies are needed to clarify the efficacy of these compounds *in vivo*. In the case of chlorpromazine, the concentration required in the intestine to remove resistance plasmids is considerable (Maier et al., 2018). For this reason, it is necessary to develop strategies that allow targeted delivery of these chemical agents and avoid oral administration in order to increase their efficiency and reduce the risk of toxicity.

### Biological Strategies for Removing MGEs

#### Bacteriophages

Bacteriophages are viral parasites capable of infecting bacteria by recognizing surface receptors, injecting their genetic material into the host, and replicating using the host cellular machinery. Phages exert ecological and genetic effects on bacteria at the population level, and these effects can impact plasmid stability (Thompson, 1994). This is due to epistatic interactions between the cost of chromosomal phage-resistant mutations and the cost of maintaining plasmids (Buckling and Rainey, 2002). Although phage-mediated transfer of ARGs between bacteria has been demonstrated for numerous bacterial species, the transduction occurs at a low rate (between 10^–6^ and 10^–9^ transductants/pfu). One exception is methicillin-resistant *S. aureus* that contains a category of MGEs called phage-inducible chromosomal islands (PICIs), which are associated with the highest transduction frequency (10^–1^ transductants/pfu) (Calero-Caceres and Muniesa, 2016; Torres-Barcelo, 2018). Phages may enhance the persistence of ARGs as an adaptation strategy to restrictive environmental conditions, e.g., wastewater aggressively treated using UV, temperature, or pH. However, genetically modified phages could be used to increase antibiotic susceptibility of resistant strains. The alarming increase in resistance has also led to the revival of phage therapy in order to sensitize resistant bacteria by eliminating resistance and virulence factors (Lin et al., 2017). Jalasvuori et al. (2011) showed that the PRD1 phage determined the loss of RP4 and RN3 resistance plasmids from strains of *E. coli* and *Salmonella* spp. and inhibited the conjugation ability of the remaining resistant bacteria. Another study demonstrated that the M13KE filamentous phage could block plasmid conjugation by interacting with the conjugative *F pilus* in *E. coli*. Furthermore, addition of the M13 phage g3p minor protein results in complete inhibition of conjugation, suggesting this protein has an essential role in the process (Lin et al., 2011). Harrison et al. (2015) eliminated the pQBR103 megaplasmid in *P. fluorescens* using the SBW252 lytic phage. Recently, Chan et al. (2016) revealed that the OMKO1 phage isolated from *P. aeruginosa* could sensitize antibiotic-resistant strains to erythromycin, ceftazidime, tetracycline, and ciprofloxacin. Together, these studies demonstrate the possibility of using phages to reduce the prevalence of resistance plasmids in bacterial populations as well as to block plasmid conjugation. In addition, phages can be successfully used to increase the sensitivity of bacterial strains to antibiotics.

Seemingly successful experimental trials using phages to treat pediatric dysentery (Summers, 2004), cholera and skin infections (Abedon et al., 2011), and bubonic plague (Summers, 2004) sparked interest in phage therapy both in Europe and the United States. However, attempts to repeat these trials and achieve positive results failed; this was due to an incomplete understanding of phage biology, and because of the large-scale development of a wide range of antibiotics that could be used to treat these infections. Experimental data obtained from the use of phage therapy in animals, as well as data from observational studies conducted in humans, were not followed by clinical studies to confirm the therapeutic value of phages. However, in recent years, the abusive use of broad-spectrum antibiotics (Ventola, 2015), as well as the rapid evolution and dissemination of resistant bacteria (Kumarasamy et al., 2010), has stimulated research into phage therapy (Lin et al., 2017), and data from promising clinical trials have been published. Schooley et al. (2017) have used phagotherapy in a patient with necrotic pancreatitis caused by a MDR strain of *A. baumannii*. Other studies have obtained favorable results for the phagotherapy of an aortic graft infection with *P. aeruginosa* (Chan et al., 2018), pneumonia caused by a MDR strain of *P. aeruginosa* in a cystic fibrosis patient (Law et al., 2019), a *Mycobacterium abscessus* infection in a patient with cystic fibrosis (Dedrick et al., 2019), and periprosthetic, musculoskeletal, and lung infections (Maddocks et al., 2019; Onsea et al., 2019; Tkhilaishvili et al., 2019). Contrary to these studies, there are reports of the inefficiency of phages in treating bacterial infections (Sarker et al., 2016; Jault et al., 2019), which suggests that the clinical use of phages requires standardization. One of the greatest challenges in phage therapy is the selection of bacterial strains that are resistant to phage action (Azam and Tanji, 2019; Taylor et al., 2019; Yuan et al., 2019). Further studies are required to clearly understand phage biology and elucidate the mechanisms leading to the emergence of phage resistance.

#### Incompatibility-Based Plasmid Curing and Toxin/Antitoxin Systems

Plasmid incompatibility is generally defined as the inability of two co-resident plasmids to be stably inherited in the absence of outside selection. Thus, if the introduction of a second plasmid destabilizes transmission of the first plasmid, the two plasmids are incompatible. This occurs because the two plasmids share the same replication and partitioning mechanisms. Consequently, under the influence of selective pressure, the resident plasmid can be eliminated (Novick, 1987). Elimination of plasmids based on incompatibility has historically been used to elucidate the mechanisms involved in elimination, and to study the interactions between the plasmid and the host (Uraji et al., 2002). One of the main disadvantages of incompatibility-based plasmid curing methods is the need for repeated cloning and detailed knowledge of the target plasmid. Also a significant problem in the construction of interference plasmids is the requirement to know the replication and partition control region before curing, as well as the need to include additional plasmid genes (Ni et al., 2008). This incompatibility-based strategy has been employed in a variety of bacteria. In *L. acidophilus*, *L. plantarum*, and *L. pentosus* it was used to eliminate approximately 2.3-kb resident plasmids (Bringel et al., 1989; Posno et al., 1991). Ni et al. (2008) used plasmid incompatibility to study the role of plasmids in the pathogenesis of *Yersinia pestis*. The technique has also been used to remove strains of *Bacillus anthracis* carrying high pathogenicity plasmids, thus allowing observation of their role in capsule formation and toxin production (Wang et al., 2011; Liu et al., 2012). Hale et al. (2010) constructed a plasmid incompatibility system called pCURE to eliminate F-like plasmids and IncP-1α from *E. coli*. This system comprises elements of repression (transcriptional repressor, antisense RNA), the origin of replication to compete for essential steps, as well as an antitoxin repressor to control the toxin/antitoxin system (Hale et al., 2010). Toxin/antitoxin systems, also known as post-segregational cell killing or addiction systems, are components of natural plasmids that ensure their persistence in bacterial populations by blocking the growth of daughter cells that do not inherit the plasmid. These systems consist of a labile antitoxin that quenches the activity of the stable toxin. Blocking antitoxin gene expression upon plasmid loss leads to faster depletion of the antitoxin than the toxin, which de-represses toxin activity, and ultimately results in programmed cell death (Hayes, 2003). Recently, Kamruzzaman et al. (2017) constructed incompatibility plasmids in combination with genes encoding antitoxins and replicons, in order to eliminate the bla_*IMP–*__4_ and bla_*CMY–*__2_ genes, in both *in vitro* and *in vivo* experimental models. Target plasmids were eliminated in the presence of antibiotics for selecting for the interference plasmid (Kamruzzaman et al., 2017).

Successful *in vitro* elimination of plasmids through incompatibility systems suggests that this strategy could be applied *in vivo*, to both humans and animals. However, in order to achieve this desideratum, in-depth research is necessary to overcome current limitations of the system, such as the need for repeated cloning, detailed characterization of target plasmids, and prior knowledge of replication and segregation control regions (Ni et al., 2008). Moreover, the requirement to use antibiotics to select eliminated plasmids may be a significant disadvantage to the method. Another aspect that needs further research is the interaction between the interference and resistance plasmids, including the reduction of antibiotic selection.

### Utilization of CRISPR/Cas System to Eliminate MGEs Involved in AMR

As stated above, strategies to remove MGEs based on chemical compounds, phages, or incompatibility based-curing plasmid systems have many limitations. All previously described strategies require several stages of bacterial growth in the presence of stressor agents, such as high temperature or intercalating agents, which may lead to unwanted mutations. Therefore, novel approaches for the elimination of MGEs involved in AMR have been proposed.

**FIGURE 3:**
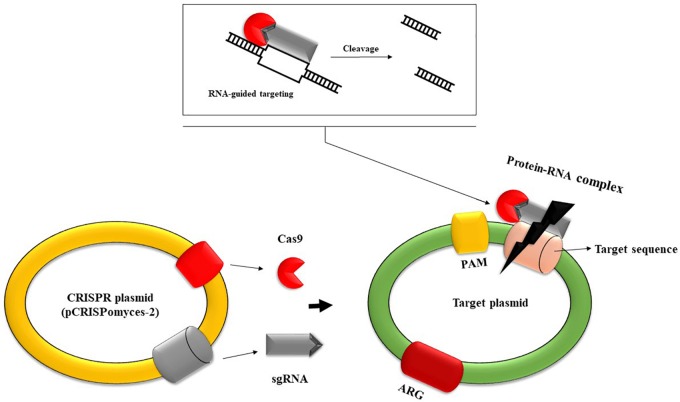
Schematic representation of CRISPR-based plasmid system capable of removing MGE-like resistance plasmids. This system contains two sgRNA transcripts, the *cas9* nuclease, and other structural elements. Firstly, sgRNA forms a complex with *cas* nuclease. The sgRNA transcripts guide *cas9* nuclease to introduce double-stranded breaks at the ends of the target DNA, leading to cleavage. Direct target recognition is achieved through recognition of protospacer adjacent motifs (PAM), short DNA sequences that are not found in CRISPR loci, so there is no risk of self-degradation (So et al., 2017). Subsequently, the gap is filled through homologous recombination by an editing template. This system can be used to edit the genome of several antibiotic-resistant bacterial strains, leading to the removal of resistance determinants.

An attractive alternative strategy for combating bacterial resistance uses the CRISPR (Clustered Regularly Interspaced Short Palindromic Repeat) system, which was initially described in 1987 by Ishino et al. (1987). CRISPR/Cas is an immune defense system in bacteria that is capable of recognizing foreign nucleic acids and destroying them through associated caspases. One of the significant advantages of this system is its high specificity. This is due to the existence of short repetitive sequences in CRISPR loci that are separated from each other by single sequences of 26–72 pairs derived from MGEs such as plasmids or transposons (Li H. Y. et al., 2018). The CRISPR/Cas mechanism of defense against foreign genetic elements is accomplished in three stages: acquisition, expression, and interference (Crawley et al., 2018). The acquisition stage comprises the insertion of single sequences (spacers) derived from MGEs into repetitive loci of the host chromosome; these sequences are separated from each other by repetitive sequences. The expression stage involves transcribing the complex of repetitive and spacer sequences into a single RNA transcript that will be further processed by caspases in short CRISPR RNAs. In the final stage, the interference phase, foreign nucleic acids are identified based on complementarity with CRISPR RNAs, and their degradation is executed by caspases (Walker and Hatoum-Aslan, 2017). Discrimination between self and non-self is accomplished through sequences from the foreign nucleic acid called protospacers. These sequences are positioned between short DNA sequences (2–6 bp) called protospacer adjacent motifs (PAMs). Cas9 (CRISPR-associated protein 9) will not cleave to a protospacer sequence unless there is a neighboring PAM. CRISPR loci do not contain PAMs, hence direct target recognition is achieved by the CRISPR system without the risk of degrading its own nucleic acid (Marraffini and Sontheimer, 2010; Figure 3). The CRISPR system is classified into six main types and 33 subtypes. Each type has several structural and functional characteristics, but the most distinctive feature is the *cas* genes and proteins they encode, which play an essential role in recognizing and degrading invading nucleic acids. The number of *cas* genes ranges from 4 to 20, and the diversity of the corresponding Cas proteins form an ensemble of properties that are essential to the CRISPR immune mechanism (Makarova and Koonin, 2015).

The idea that the CRISPR system could acquire new repetitive nucleic acid sequences of extrachromosomal origin, mainly from phages and plasmids, significantly increased interest in using the system to limit HGT by blocking plasmid conjugation (Bolotin et al., 2005; Pourcel et al., 2005). Subsequently, there have been numerous studies on this topic. The first study of this type was conducted by Marraffini and Sontheimer (2008). The authors attempted to use the CRISPR system interference to block the conjugation of plasmids in *S. epidermidis*. *S. epidermidis* strains contain a CRISPR locus containing a homolog *spc1* spacer with a region encoding the nickase gene found in conjugative plasmids from this strains (Diep et al., 2006). Silent mutations were introduced into the target gene of the pG0400 conjugate plasmid, generating a mutant plasmid (pG0). Both wild-type and mutant plasmids were tested for conjugation ability. In the control strain, which lacked the CRISPR locus and *spc1* complementary to the nickase gene, the conjugation frequency was similar for both plasmids. In the strain harboring the CRISPR locus, only the mutated plasmid with the modified sequence was transferred by conjugation. This demonstrated that the CRISPR system could block plasmid conjugation in a site-specific manner. Furthermore, based on the complementarity between the *spc1* spacer and the nickase gene region, the authors showed that CRISPR interference could block plasmid transformation (Marraffini and Sontheimer, 2008). Jiang et al. (2013) showed that the CRISPR system could edit the genome of *E. coli*. An A to C transversion was introduced in the *rpsL* gene and a pCRISPR:*rpsL* plasmid harboring a spacer that would guide dual-RNA:Cas9 cleavage of the wild-type *rpsL* gene was constructed. Following incubation of the strain of interest with the plasmid, deletion of the *rpsL* gene was observed (Jiang et al., 2013). Removal of ARGs was also demonstrated by Citorik et al. (2014), using a variant of the CRISPR system encountered in *S. pyogenes*. The authors built plasmids in which they introduced the CRISPR elements as well as a copy of the *bla*_*SHV–*__18_ and *bla*_*NDM–1*_ target genes, conferring resistance to extended-spectrum beta-lactam antibiotics. Elimination of *bla*_*SHV*_*_–_*_18_ and *bla*_*NDM–1*_ plasmids was achieved by packaging the CRISPR elements into a bacteriophage. Following treatment of clinical isolates of *E. coli* bearing the target plasmids with the constructed phage, a significant reduction in viable bacterial cells was observed (Citorik et al., 2014). Removal of kanamycin resistance genes through the CRISPR system has been demonstrated by Bikard et al. (2014) for strains of *S. aureus*.

Yosef et al. (2015) introduced the CRISPR elements (*cas* genes, and spacer sequences targeting the *bla*_*NDM-1*_ and *bla*_*CTX-M-15*_ genes) into a lysogenic phage, and following lysogenization of the resistant bacteria with the constructed phage, elimination of resistance plasmids was observed. The CRISPR system had exhibited the ability to not only remove resistance plasmids, but also to block their HGT (Yosef et al., 2015). Kim et al. (2016) designed a CRISPR plasmid capable of recognizing the *bla*_*TEM*_ and *bla*_*SHV*_ genes from strains of *E. coli* producing extended-spectrum β-lactamases. Following transformation of bacterial cells with the CRISPR plasmid, elimination of plasmid-encoding beta-lactamase production was observed, demonstrating the action of caspase at the level of the *bla*_*TEM*_ and *bla*_*SHV*_ target regions. Furthermore, after elimination of the resistance plasmid, the bacterial strains became sensitive to a series of other antibiotics to which they have previously shown resistance (Kim et al., 2016). Wang et al. (2019) constructed a pMBLcas9 plasmid expressing Cas9, used to clone target single-guide RNAs (sgRNAs) for plasmid curing. The recombinant plasmid pMBLcas9-sgRNA was transferred by conjugation into two clinical isolates of *E. coli*. In this study, four native plasmids in isolate 14EC033 and two native plasmids in isolate 14EC007 were successfully eliminated in a stepwise manner using pMBLcas9. In addition, two native plasmids in 14EC007 were simultaneously eliminated by tandemly cloning multiple sgRNA in pMBLcas9, sensitizing isolate 14EC007 to polymyxin and carbenicillin (Wang et al., 2019). In *Zymomonas mobilis* strain ZM4 the resistance plasmids pZZM402 and pZZM403 were eliminated by targeting the replicase-encoding gene which, once inactivated, impairs plasmid replication and leads to subsequent elimination (Cao et al., 2017). The CRISPR system was also used to target some conserved regions within the ColE1 replicons encountered in 91% of the plasmids found in the databases. Lauritsen et al. (2017) constructed a vector in which they introduced all required CRISPR elements as well as two complementary RNA sequences with the conserved regions that guide the cascade nine nuclease to introduce double-strand breaks. This induced elimination of resistance plasmids in *E. coli* and other bacteria possessing replicons with conserved regions that are targets for the CRISPR system (Lauritsen et al., 2017). CRISPR systems have been designed and delivered in *E. coli* via transformation and conjugation to eliminate the plasmid-borne *mcr-1* gene (Sun et al., 2017; Dong et al., 2019). Efficient editing of a target locus using a CRISPR-based system was achieved in *S. aureus* (Liu et al., 2017), *B. subtilis* (So et al., 2017), *E. faecalis* (Hullahalli et al., 2017), and *E. coli* (Lauritsen et al., 2018). In summary, this array of studies conducted on various bacterial strains demonstrates the utility of the CRISPR system to eliminate resistance plasmids, as well as blocking HGT of the plasmids. The CRISPR system can also be used for antibiotic sensitization of resistant strains.

Many protocols for plasmid curing/ARG elimination using CRISPR have been proposed (Table 3). However, there are some limitations of this strategy. These limitations include: a known target plasmid replication mechanism is required,; there is a risk of chromosomal ARG acquisition in the interfering plasmid (Kamruzzaman et al., 2017); and the majority of the studies demonstrating the ability of the CRISPR system to eliminate resistance plasmids, as well as to block the dissemination of ARGs by HTG, were performed *in vitro*. The efficiency of the CRISPR system to eliminate ARGs has been demonstrated *in vivo* in different mammalian models. Price et al. (2019) revealed that the *E. faecalis* CRISPR system could block dissemination of resistance plasmids in the mouse gut. However, it remains to be established how much can be extrapolated from these studies to other mammalian organisms. Successful oral administration of phages for targeting bacteria in the intestinal tract (Corbellino et al., 2019) has led to the proposal that phages could be used as a vehicle for delivering the CRISPR system into intestinal microbiota to eliminate ARGs. However, this would require a collection of phages specially designed to target ARGs, the optimal concentration would need to be established, and knowledge of several barriers that occur *in vivo* would be required, such as inactivation of bacteriophages by gastric acid, and neutralization of phages by the spleen and the immune system (Merril et al., 2003).

**TABLE 3:** Protocols used for plasmid curing/ARGs elimination via CRISPR.

Curing/Elimination strategy	Species of interest	Plasmid/Genes target	Delivery	Results	References
Plasmid conjugation blocking by CRISPR system via complementarity between *spc*1 CRISPR locus and a *nes* gene region from staphylococcal plasmids	*S. aureus S. epidermidis*	pG0400 plasmid *nes* gene	Conjugation Electroporation	Plasmid conjugation and transformation blocking Limiting the spread of antibiotic resistance	Marraffini and Sontheimer, 2008
*S. pneumoniae*: introducing the *erm*AM gene together with a premature stop codon in the *srt*A locus. *E. coli* pCRISPR:rps*L* plasmid construct with a spacer that would guide Cas*9* cleavage	*S. pneumoniae E. coli*	*erm*AM, erythromycin resistance gene *rpsL*, streptomycin resistance gene	Transformation	Killing of transformed cells CRISPR selection of non-edited cells	Jiang et al., 2013
CRISPR plasmid constructs bearing the copy of target genes, introduction of double-strand breaks by complementary RNA-guided nucleases in targets CRISPR bacteriophage constructs using for targeting plasmids of interest	*E. coli Galleria mellonella*	*bla*_SHV–18_ *bla*_*NDM–1*_ *pZE-bla_*NDM–1–gfp*_*	Conjugation Viral transduction	Sequence-specific cytotoxicity Excluding high-copy antibiotic resistance plasmids Re-sensitizing a resistant population to antibiotics.	Citorik et al., 2014
Insertion of CRISPR array in a staphylococcal vector to obtain pDB114, programmed to target kanamycin resistant gene Antimicrobial CRISPR cas phagemid to target the methicillin resistance gene CRISPR array that target plasmids of interest	*S. aureus*	*aph*-3, kanamycin resistance gene *mec*A, methicillin resistance gene pUSA01 pUSA02 pUSA03	Transformation Transduction	Sequence-specific killing of staphylococci resistant to kanamycin or methicillin Loss of pUSA02 plasmid Immunization of staphylococci against pUSA02 transfer.	Bikard et al., 2014
Phage transferable CRISPR cas system	*E. coli*	*bla*_*NDM–1*_ and *bla*_*CTX–M–15*_ encoding resistance to carbapenems *pNDM* and *pCTX* antibiotic resistance plasmid	Lysogenization Transformation	Resistance plasmid curing Prevention of horizontal gene transfer Sensitizing bacteria to multiple antibiotic resistance genes	Yosef et al., 2015
CRISPR plasmid construct, able to recognize target sequences from ESBL strains	*E. coli*	*bla*, beta-lactamase genes *tet*, tetracycline resistance gene pUC19 mediating Amp resistance pET21b pBR322	Transformation Conjugation	Re-sensitization to antibiotics of *E. coli* carrying ESBL plasmids CRISPR mediated clearance of whole plasmids	Kim et al., 2016
CRISPR plasmid designed to target a sequence from the replicase gene	*Zymomonas mobilis*	pZZM402 pZZM403	Transformation Electroporation	Elimination of native plasmid of *Z. mobilis*	Cao et al., 2017
Targeting conserved regions from colE1 replicons via CRISPR	*E. coli P. putida*	pZE-GFP, pZA-GFP pZS-GFP	Transformation	Efficient plasmid curing	Lauritsen et al., 2017
*mcr*-1 knockout via pCas: mcr CRISPR plasmid	*E. coli*	Plasmid-borne *mcr*-1 gene, colistin resistant.	Electroporation BMAP-27 antimicrobial peptide	Sensitization of *E. coli* strains to colistin following *mcr*-1 elimination	Sun et al., 2017
pLQ-Pxyl/tet-cas9-Pspac-sgRNA construct, designed to target plasmid of interest	*S. aureus*	pLQ-KO-tgt-50 bp pLQ-KO-rocA	Transformation	Efficient editing of the target locus	Liu et al., 2017
CRISPR -based plasmid pHCas9 targeting pHT01 and pB0A	*B. subtilis*	pHT01 pB0A	Transformation	Both plasmids cured by serial culture in antibiotic-free conditions	So et al., 2017
CRISPR2 locus manipulation to obtain pCR2-ermB and pCR2-Phage1	*E. faecalis*	PRP pTEF1 pAM771	Conjugation Electroporation	Decrease in conjugation frequency of the plasmids harboring ARGs	Hullahalli et al., 2017
CRISPR Cas *9* – based plasmid curing system (pFREE) targeting all major plasmid replicon in molecular biology	*E. coli*	SEVA vectors	Transformation	Efficient curing of target plasmids	Lauritsen et al., 2018
pMCas*9*- *mcr*-1 CRISPR construct able to eliminate *mcr*-1 gene pMob-Cas*9* construct, delivered by conjugation	*E. coli*	Plasmid-borne *mcr*-1 gene, conferring colistin resistance	Transformation Conjugation	Elimination of plasmid-borne *mcr*-1 via CRISPR system delivered via transformation and conjugation assay	Dong et al., 2019
Metal stressors exposure	*E. coli*	pKJK5 broad range plasmid	Conjugation Filter mating experiments	Plasmid elimination	Klumper et al., 2017

## Conclusion

The global increase in antibiotic resistance is a significant challenge in the fields of medicine and microbial ecology. Rapid development of effective strategies to reduce and control bacterial resistance is required. MGEs have a pivotal role in the acquisition and transmission of ARGs in clinical and environmental sectors, and one approach to control resistance is through elimination of these MGEs. Different chemical (biocides, nanoparticles, antibodies) and biological (engineered phages, commensal microbiota) strategies have been developed, with most of the strategies being directed toward curing the resistance plasmids or inhibiting the conjugation process. However, despite the potential array of approaches directed toward elimination of MGEs, these strategies need refining to overcome the challenges identified in this literature survey. These challenges include the cost-efficiency ratio, the narrow bacterial host spectrum, resistance to phages or chemical agents, the need for a known target plasmid replication mechanism, the risk of chromosomal ARG acquisition in the interfering plasmid (CRISPR technology), and the inability to remove big plasmids. Future work should focus on tackling these challenges to develop a successful strategy to combat antibiotic resistance.

## Author Contributions

MC conceived and corrected the manuscript. MC, CB, LP, and CV contributed to the literature survey and revised the manuscript. CV drafted the manuscript. LP designed the figures.

## Conflict of Interest

The authors declare that the research was conducted in the absence of any commercial or financial relationships that could be construed as a potential conflict of interest.
